# An unusual phaeoid fungi: *Ulocladium*, as a cause of chronic allergic fungal sinusitis

**Published:** 2010-06

**Authors:** R Kaur, A Wadhwa, A Gulati, AK Agrawal

**Affiliations:** 1 Department of Microbiology; 2 Department of Otorhinolaryngology, Maulana Azad Medical College, New Delhi, India

**Keywords:** Ulocladium, Phaeoid Fungi, Chronic Sinusitis

## Abstract

Allergic fungal sinusitis (AFS) has been recognized as an important cause of chronic sinusitis commonly caused by *Aspergillus* spp. and various dematiaceous fungi like *Bipolaris*, *Alternaria*, *Curvalaria*, and etc. *Ulocladium botrytis* is a non pathogenic environmental dematiaceous fungi, which has been recently described as a human pathogen. *Ulocladium* has never been associated with allergic fungal sinusitis but it was identified as an etiological agent of AFS in a 35 year old immunocompetent female patient presenting with chronic nasal obstruction of several months duration to our hospital. The patient underwent FESS and the excised polyps revealed *Ulocladium* as the causative fungal agent.

## INTRODUCTION

Chronic allergic sinusitis is a common condition responsible for the development of nasal polyps, described as abnormal lesions that emanate from any portion of the nasal mucosa or Para nasal sinuses. They are commonly located in the middle meatus and ethmoid sinus and are present in 1–4% of the population (1). Nasal polyps form in response to continuous long term inflammatory and infectious stimuli (2). Allergic fungal sinusitis is a form of chronic non invasive sinusitis which has recently gained importance. It is usually caused by *Aspergillus* spp, and few dematiaceous fungi like *Curvularia*, *Alternaria* and *Bipolaris* (3). We present a case of chronic allergic fungal sinusitis with bilateral nasal polyps due to an unusual phaeoid fungus, Ulocladium botrytis.

## CASE REPORT

A 35 year old female patient presented to the ENT out patient department of the Lok Nayak Hospital, Delhi, with chronic nasal obstruction, excessive sneezing, nasal discharge and frontal headache since several months. Nasal obstruction was of gradual onset, non progressive, more on the left side than right. Nasal obstruction did not respond to standard medical treatment and was of recurrent nature. The nasal discharge was intermittent, purulent and nonfoul smelling. There was no history of hemifacial pain, ear discharge, ear ache, nasal trauma or previous nasal surgery. No history of any allergies to drugs or food products was present. Systemic history was uneventful.

On examination the nose showed external parrot beak deformity and there was mild external deviation of the nose to the right. Nasal septum was deviated to right inferiorly, and towards left superiorly. There was Candal deviation to left. Nasal patency was decreased on the right side and Cottle's test was positive. No sinus tenderness could be elicited.

**Fig. 1:**
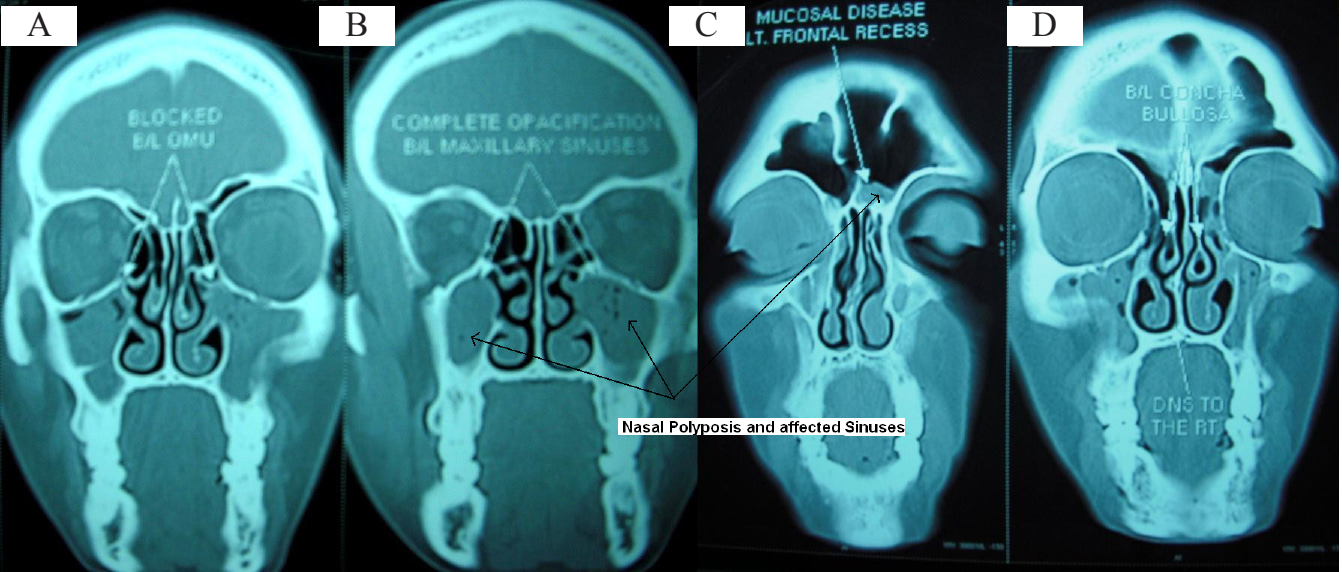
Coronal sections at various planes showing (A) bilateral blocked osteo-meatal units, (B) complete opacification of bilateral maxillary sinuses, (C) mucosal disease of left frontal recess and (D) bilateral concha bullosa & DNS to the right.

Differential leukocyte count of the patient showed eosinophilia suggesting an allergic etiology and the Non Contrast CT PNS revealed a deviated nasal septum towards right, bilateral concha bullosa, mucosal thickening in left frontal recess, left anterior ethmoidal air cells and right sphenoidal sinus and bilateral inferior turbinates. Near total opacification in both maxillary sinuses with blocked osteomeatal units were seen (Fig. 1). There was no bony erosion or invasion on CT scan. The major CT feature of allergic sinusitis is the presence of a soft tissue mass within the involved sinus, seen on unenhanced studies (4). Failing any improvement on medical therapy, bilateral fibreoptic endoscopic sinus surgery with septoplasty was planned under GA and the biopsy was excised and sent for fungal culture.

**Fig. 2:**
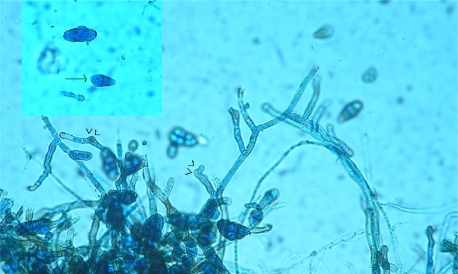
Micrographs of the isolate prepared from a 5 day old culture and stained with lactophenol cotton blue showing muriform verrucose conidia with geniculate (bent) conidiophores (arrowheads) and (inset) showing oblique septation in a conidium indicated by an arrow (magnification, ×400)

Both Gram staining of the impression smears of the tissue crushed between two sterile slides and 10% KOH preparation showed the presence of fungal hyphae. Biopsy material was inoculated on a set of Sabouraud dextrose agar with and without antibiotics and one tube of each set was incubated at 22°C and 37°C respectively. On the 5th day of incubation, a black colored wooly growth was seen in all four tubes. The LPCB preparation of the growth showed phaeoid septate hyphae with internodal branching and macroconidia with alternate septations similar in morphology to Alternaria, but the presence of verrucose, muriform macroconidia borne from short geniculate conidiophores showing 2-3 transverse septa and 1-2 longitudinal and/ or oblique septation indicated towards *Ulocladium* spp. A slide culture was done and the absence of chains of macroconidia ruled out *Alternaria* spp. and confirmed the fungi to be *Ulocladium botrytis* morphologically (Fig. 2).

## DISCUSSION

Fungal rhinosinusitis can be broadly divided into two categories: the invasive and non-invasive depending on invasion of the mucosal layer. Three types of FRS are tissue-invasive: acute invasive, chronic invasive, & granulomatous. The two noninvasive FRS disorders are fungal ball, and fungus related eosinophilic rhinosinusitis including allergic fungal rhinosinusitis (AFRS) (5). Allergic fungal sinusitis affects healthy and immunocompetent young adults. Patients are usually atopic, often having h/o asthma and nasal polyposis as seen in this case. The diagnostic criteria for Allergic fungal sinusitis include type 1 hypersensitivity, nasal polyposis, characteristic CT scan, histological evidence of eosiniophilic mucous without evidence of fungal invasion into the sinus tissue and/or a positive fungal stain or culture from the sinus (6). Our case seemed to fulfill the definition, with characteristic involvement of multiple sinuses. Most fungi implicated in AFS are typically cultures positive for either dematiaceous fungi such as *Bipolaris spicifera* or *Curvularia lunata*, or *Aspergillus* species such as *A. fumigatus,A. flavus* or *A. niger* (3, 7–9) with *Aspergillus* spp. being more common in India and no case of chronic sinusitis due to *Ulocladium* has been reported from any part of the world.

*Ulocladium*, previously considered an environmental fungus with no pathogenic potential, has been implicated in various mycotoxicoses due to ingestion of infected wheat etc and has been most commonly associated with plant mycology (10, 11). Recently there have been case reports associating *Ulocladium* with various types of infections, both in immunocompetent patients as well as in immunocompromised. *Ulocladium* spp. has been shown to cause keratitis (12), and onychomycosis, (13), in immunocompetent adults while cutaneous infection has been reported in some immunosuppressed patients (14, 15). It has been understood for a very long time that environmental fungi play an important role in the pathogenesis of AFS but till now no case has been reported implicating *Ulocladium* as a causative agent. This report is thus the first of its kind to show that other non pathogenic dematiaceous fungi like *Ulocladium botrytis* may also be responsible for the occurrence of Allergic fungal sinusitis and nasal polyposis.
